# Cardiometabolic profile of obese children in a sub-Saharan African setting: a cross-sectional study

**DOI:** 10.1186/s12887-017-0880-2

**Published:** 2017-05-19

**Authors:** Eunice Chedjou-Nono, Suzanne Sap, Simeon-Pierre Choukem, Issa Ngosso Tetanye, Daniel Nebongo, Olivier Koki Ndombo

**Affiliations:** 1 Higher Institute of Health Sciences, Université des Montagnes, Bangangte, Cameroon; 2 Faculty of Medicine and Biomedical Sciences, University of Yaounde I, Yaounde, Cameroon; 3 Mother and Child Center of the Chantal BIYA Foundation, Yaounde, Cameroon; 4 Health and Human Development (2HD) Research Group, Douala, Cameroon; 5 Diabetes and Endocrine Unit, Department of Internal Medicine, Douala General Hospital, Douala, Cameroon; 6 Department of Internal Medicine and Pediatrics, Faculty of Health Sciences, University of Buea, Buea, Cameroon; 7 Frantz Fanon Center, Yaounde, Cameroon

## Abstract

**Background:**

Cardiovascular and metabolic consequences of obesity in children, unlike adults, are still not well understood nor have they been subject to extensive research in Africa. We aimed to identify the cardio-metabolic complications associated with childhood obesity at the early phase of the management of obese children in a reference center in Cameroon.

**Methods:**

In this cross-sectional study conducted from November 2013 to September 2014 and based on World Health Organization (WHO) classification of Obesity (BMI > 3SD under 5 years and BMI > 2SD from 5 and above), we included children aged 3 to 17 years who were being followed up for obesity at the pediatric endocrinology unit of the Mother and Child Center of the Chantal BIYA Foundation in Yaounde, Cameroon. A control group composed of children with normal BMI coming for a routine check up or vaccination was matched to the obese subjects. In both groups, we measured waist circumference (WC), blood pressure, fasting lipid profile and fasting glycaemia. We also considered the presence or absence of acanthosis nigricans. Data were analyzed using STATA software version 11.0, and presented as means, medians, compared with parametric and non-parametric statistical tests.

**Results:**

We enrolled 38 obese children and 38 controls matched for sex and age. The majority of our participants were boys with a sex ratio of 1.24, and median age was 9.9 years. The median Z score of BMI was 3.21 in obese children. Approximately (*n* = 35) 90% of obese children (<6% in controls *p* < 0.001) presented with an abdominal obesity (WC/height ratio > 0.5) and 58% (*n* = 22) had acanthosis nigricans (5% (*n* = 2) in controls, *p* < 0.001). Type 2 diabetes mellitus was found in one participant, hypercholesterolemia in about 16% (*n* = 6) and high blood pressure in 25% (*n* = 8) of participants. Metabolic syndrome was present in 19% (*n* = 4) of obese children aged >10 years.

**Conclusions:**

Obesity in children is associated with early onset metabolic disorders such as dyslipidemia, high blood pressure and type 2 diabetes. The screening and management of these complications is therefore recommended.

## Background

In the past few decades, obesity has been on a sharp increase in children and adolescents [1]. According to WHO statistics, there were about 43 million obese children worldwide in 2011 with 35 million of them living in developing countries [2]. There is a paucity of data on childhood obesity in Sub-Saharan Africa. Notwithstanding, in Cameroon, the 2011 Health and Demographic Survey (DHS) revealed that 6% of children below 5 years were obese [3], and 10 to 15% of adolescents were affected in urban areas [4–6].

Early and late complications of childhood obesity are numerous and severe. They include metabolic diseases [7], cardiovascular diseases [8–11], bone deformities and respiratory problems [1, 12]. Moreover, childhood obesity is likely to persist throughout adulthood, thereby exposing obese subjects to cardiovascular, metabolic, respiratory, musculoskeletal and psychosocial problems. And such illnesses may lead to an increased morbidity and mortality in this population [9].

There is currently very few data on the metabolic status of obese children in sub-Saharan Africa. Therefore, the aim of this study was to identify cardio-metabolic abnormalities in obese children who attended the pediatric endocrinology outpatient clinic of the Mother and Child Centre of the Chantal BIYA Foundation in Yaounde, Cameroon, comparing them with matched children with normal Body Mass Index (BMI).

## Methods

### Study design, setting and participants

This cross-sectional study received ethical approval from the institutional ethical committee of “Université des Montagnes” (number 2014/047/UdM/PR/CAB/CIE). Participants were included from the pediatric endocrinology unit of the Mother and Child Centre of the Chantal BIYA Foundation in Yaoundé from November 2013 to September 2014. The Center is a multidisciplinary pediatric hospital located in the capital and second most populous city of Cameroon; it hosts the only pediatric endocrinology unit of the country. This unit allocates two separate rooms to metabolic investigations.

#### Selection of cases

We carried out a consecutive sampling of children and adolescents aged 2 to 17 years with obesity defined according to WHO recommendations as a body mass index (BMI) > 3 Z-score for children below 5 years and BMI > 2 Z-score for those from 5 years and above [13].

#### Selection of control subjects

The control group was made up of children with normal BMI (−2 Z-score < BMI < +1 Z-score). They were selected among healthy children who came for a routine checkup or vaccination and were matched with patients for age and sex.

### Data collection and definition of terms

The waist circumference (WC) in centimeter (cm) was measured with a non-stretchable tape at a point midway between the costal margin and the anterior superior iliac spine, and the standing height (H) in cm with a wall-mounted stadiometer. The weight in kilogram (kg) was measured with a Seca® brand bathroom scale. The BMI was calculated as the weight in kg divided by the square of the height in meter. Abdominal adiposity was defined as waist-to-height ratio (WHtR = the waist circumference in cm divided by the height in cm) ≥ 0.5.

The blood pressure (BP) was measured at rest with an appropriate cuff for each child with a manual Spengler® sphygmomanometer, and the average of three measures taken at 10 min intervals was considered and then translated in percentile with the Baylor College of Medicine software [14]. Blood pressure was classified according to recommendations of the American Academy of Pediatrics as follows: hypertension for a BP ≥ 95th percentile, pre-hypertension for BP between the 90th and the 95th percentile [15]. The presence or absence of acanthosis nigricans was used as the insulin resistance indicator.

Parameters of the fasting lipid profile (total cholesterol, triglycerides, High Density Lipoprotein (HDL)-cholesterol, and Low Density Lipoprotein (LDL)-cholesterol calculated by the Friedewald formula) were measured in a 2 ml blood sample using a dry chemistry method in a Vitros® 350 automaton from Ortho Clinical Diagnosis, USA. Lipids abnormalities were defined according to age, following the recommendations of the American Academy of Pediatrics [16]. Fasting plasma glucose level was measured by the colorimetric method in the Vitros® 350 automaton. Diabetes was defined as a fasting plasma glucose ≥126 mg/dl (7 mmol/l). Impaired fasting glucose (IFG) was defined as a fasting plasma glucose level between 100 and 126 mg/dl [17]. Metabolic syndrome was defined according to International Diabetes Foundation (IDF) as an association of at least 2 metabolic disorders to abdominal obesity [18].

All control subjects underwent similar physical assessments with measurement of anthropometric parameters and blood pressure. Point-of-care devices were used to measure their biochemical parameters: cardio Check® (Polymer Technology System, USA) for capillary lipid profile analysis [19], and Accu Chek Active® (Roche Diagnostics GmbH, Germany) for capillary blood glucose [20].

### Ethical approval

Before inclusion into the study, a written informed consent was signed by parents of all participants, and children above 12 years gave their assent. We also obtained ethical approval from the institutional ethical committee of “Université des Montagnes” (number 2014/047/UdM/PR/CAB/CIE).

### Statistical analysis

Data were analyzed with STATA® software version 11.0 (Lakeway Drive, Texas, USA). Results were presented as mean (standard deviation: SD) for quantitative data and median (interquartile range: IQR) for qualitative data. The chi-square test was used for comparison of categorical variables. The CardioCheck® device has a lower value detection threshold for quantitative analysis; hence we considered this lower value in controls whose values were under that threshold. We used non parametric tests when variances were not homogeneous. Statistical significance was set at *p* value <0.05.

## Results

### General characteristics of participants

We included 38 out of 42 eligible participants (4 participants were excluded due to incomplete data) who were receiving care in the unit, 21 of whom were boys (55.5%). The mean age was 9.9 years (range 3–17 years). Table 1 summarizes the general characteristics of study participants and control subjects.

**Table 1:** General characteristics of study participants

Variable	Obese	Control	*P* value
N	38	38	
Boys N (%)	21 (55.3)	21 (55.3)	
Age (years)^a^	9.7 ± 3.6	10.1 ± 3.7	0.729ǂ
Weight (Kg)^a^	65.6 ± 13.7	35.2 ± 27.5	<0.0001ǂ
Z-score-Height	1.27 [0.29 - 1.91]	−0.15 [−0.74 - 0.60]	<0.0001¶
BMI (Kg/m^2^)^a^	29.3 ± 7.3	17.4 ± 2.4	<0.0001¶
Z score-BMI^b^	+3.3 [2–7]	0 [−1.5 + 1]	<0.0001¶
WC (cm)^b^	88.5 [80.0-96.5]	61.0 [56.0-65.0]	<0,0001¶

^a^mean ± SD; ^b^ median [IQR]; ǂ t-test; ¶ Man-Whitney test

### Cardiometabolic profile

**Fig. 1:**
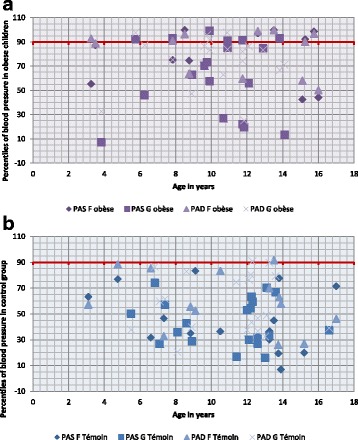
**a** Representation of percentiles of blood pressure in patients. **b** Representation of percentiles of blood pressure in controls

Out of the 31 participants who had blood pressure values during the first consultation, 14 (45%) had high values (>90th percentile).; among these, 8 (25% of all participants) had hypertension (>95th percentile): 5 girls and 3 boys, all aged above 10 years and 6 had pre-hypertension(Fig. 1a). In the control group, 2 subjects had pre-hypertension (BP between the 90th and 95th percentile) but none had hypertension (BP > 95th percentile) (Fig. 1b). The difference in prevalence of abnormal blood pressure was significant between the two groups (*p* < .0001).

**Fig. 2:**
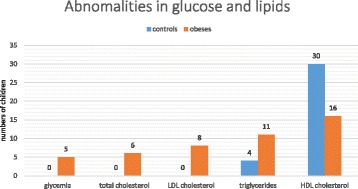
Histogram showing the numbers of children who had abnormal glucose and lipid levels

The median fasting blood glucose level (87 mg/dl) was similar in the two groups. However, one obese child was diagnosed of Type 2 diabetes mellitus and 4 children were diagnosed of IFG whereas there was none in the control group (*p* = 0.054) (Fig. 2). Compared to control subjects, obese children had higher WHtR, higher systolic and diastolic blood pressures, higher total cholesterol, LDL cholesterol, and HDL cholesterol levels (Table 2). The proportion of children with high total cholesterol, triglycerides and LDL cholesterol was higher in the obese than in the control group, whereas the proportion of children with lower HDL cholesterol level was higher in the control group (Fig. 2). Acanthosis nigricans lesions were significantly more prevalent in patients (22/38) than in control subjects (2/38) (*p* < 0.00001).

**Table 2:** Cardio metabolic status in the study population

Variable	Obese	Control	*P* value†
WHtR^a^	0.59 [0.52–0.65]	0.44 [0.42–0.46]	0.0001
SBP (mmHg)^a^	110.0 [102.0–126.0]	100.0 [96.0–110.0]	0.002
DBP (mmHg)^a^	73.3 [68.0–79.0]	64.1 [60.0–70.0]	0.05
Glycemia (mg/dl)^a^	89 [83.7–91.0]	85.5 [79.5–93.0]	0.68
Total cholesterol^a^	164.5 [142.7–187.7]	115.0 [100.0–136.3]	0.0001
Triglycerides (mg/dl)^a^	81.0 [61.0–111.0]	61.5 [50.0–71.0]	0.08
LDL cholesterol (mg/dl)^a^	102.5 [81.5–127.5]	72.0 [64.8–85.3]	<0.0001
HDL cholesterol (mg/dl)^a^	42.0 [33.8–52.0]	28.0 [24.0–36.3]	0.04

^a^median [IQR]; † Man-Whitney test

**Fig. 3:**
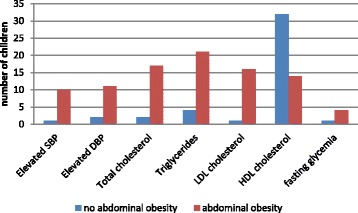
Number of children with metabolic abnormalities with respect to the abdominal obesity status

Excluding HDL cholesterol level, all other metabolic abnormalities were associated with the presence of abdominal obesity (Fig. 3). Out of the 21 obese children aged >10 years, 4 (19%) met the criteria of definition of metabolic syndrome while there was none in the control group.

## Discussion

In this study, we have found a higher prevalence of elevated blood pressure, dyslipidemia, and blood glucose abnormalities, as well as higher occurrence of acanthosis nigricans in obese children compared to their age and sex-matched lean counterparts.

Childhood obesity has been on a steep rise worldwide for the past few decades and the likelihood that this population of obese children becomes obese during adulthood is very high [9]. This phenomenon has affected developed as well as developing countries, Cameroon inclusive. Recent studies carried out in Cameroon have reported an ever-increasing prevalence of childhood obesity [3–6]. It has been observed that childhood obesity is associated with numerous and often severe short-term and long-term complications including cardiovascular, metabolic, respiratory, musculoskeletal and psychosocial problems [7–11]. However, obesity is considered as a sign of healthy living, beauty and wealth in many African cultures. As such, most parents do not consider taking their obese children to hospital for a medical check-up and follow-up [21].

The prevalence of hypertension in our study was one out of four children and about 20% had pre-hypertension. A study carried out in Bolivia found a similar prevalence, though only the systolic blood pressure was considered [22]. But another study carried out in Thailand found a lower prevalence of hypertension in obese children (20.2%) [23]. Studies have shown that the prevalence of hypertension is higher in people of African ancestry than in Asians and Europeans [24]. The difference observed is probably due to this genetic predisposition of people of African ancestry. Also, only two children in the control group had pre-hypertension (~6%). This is evidence to suggest that the high prevalence of elevated blood pressure (about 1 in 4 children) was related to obesity in these children.

Blood glucose impairment was found in 13% of obese children in our study population whereas none of the children in the control group were affected. This is further evidence to suggest that obesity in these children predisposed them to cardiometabolic abnormalities. Studies carried out elsewhere found 10.5% and 17.8% of blood glucose impairment in Thailand [23] and United States of America [25] respectively. The difference with the United States National cohort study could be due to the fact that we did not carry out an oral glucose tolerance test, thereby probably ignoring some cases of glucose intolerance.

The proportion of children with high total cholesterol, triglycerides and LDL cholesterol was higher in the obese than in the control group. This is in line with other metabolic problems which were found in this group such as glucose impairment and the presence of acanthosis nigricans (seen in about 60% of obese children compared to only about 5% in the control group). Acanthosis nigricans is a strong indicator of insulin resistance which is a very important element in the development of Type 2 diabetes mellitus. This study identified a case of Type 2 diabetes and the evidence will suggest more cases of Type 2 diabetes in this group in the near future, especially if adequate measures are not put in place to combat these complications of childhood obesity. However, we were surprised to find that the proportion of children with lower HDL cholesterol level was higher in the control group. This may suggest either the need to redefine normal standard values ​​in children, or that the rapid test we used may not have enough accuracy and specificity to be adapted in a pediatric population.

Moreover, the cardiometabolic complications observed were mostly related to abdominal obesity. Therefore, abdominal obesity measured by the WHtR could be suggested as a quick method of identification of children with high cardiovascular risk [25].

A bigger sample is however necessary to test sensitivity and specificity. We have also studied an essentially urban sample and the results could be different with a less sedentary rural population.

We acknowledge the following potential limitations in our study: we used different laboratory analyses in testing lipid profile and blood glucose in the two groups under study but some evidence showed the accuracy of rapid photometry methods (used in control groups in our study) in the follow-up of patients affected with diabetes or dyslipidemia [19, 20].

## Conclusion

Metabolic disturbances are prevalent in obese children in Yaounde, as compared to those with normal BMI. One quarter of obese children have high blood pressure, 21% of dyslipidemia and 13% of glucose intolerance with a case of type 2 diabetes. The metabolic syndrome affects 19% of our adolescents and girls seem to be more at risk. The various abnormalities are associated with the presence of abdominal obesity. Prevention should be emphasized not only on the occurrence of complications, but better still on the occurrence of obesity in children, given the cardiovascular and metabolic risk and the heavy socio-economic repercussion in developing countries such as Cameroon.

## Abbreviations

BMI: Body Mass Index
BP: Blood Pressure
HDL: High Density Lipoprotein
IDF: International Diabetes Foundation
IFG: Impaired Fasting Glucose
LDL: Low Density Lipoprotein
WC: Waist Circumference
WHO: World Health Organization
WHtR: Waist to Height Ratio
